# Thermoresponsive nanoparticles for targeted and controlled delivery of doxorubicin in triple negative breast cancer: a 2D and 3D in vitro evaluation

**DOI:** 10.1007/s13346-025-01930-9

**Published:** 2025-07-28

**Authors:** Tiago P. Ribeiro, Francisca L. Gomes, Rui Vilarinho, Christiane Salgado, Maria Cristina L. Martins, Joaquim Agostinho Moreira, Fernando J. Monteiro, Marta S. Laranjeira

**Affiliations:** 1 i3S-Instituto de Investigação e Inovação em Saúde, Universidade do Porto, Rua Alfredo Allen 208, Porto, 4200-135 Portugal; 2 INEB-Instituto de Engenharia Biomédica, Universidade do Porto, Rua Alfredo Allen 208, Porto, 4200-135 Portugal; 3 FEUP-Faculdade de Engenharia, Universidade do Porto, Rua Dr. Roberto Frias, s/n, Porto, 4200-465 Portugal; 4 Porto Comprehensive Cancer Center Raquel Seruca (P.CCC), R. Dr. António Bernardino de Almeida, Porto, 4200-072 Portugal; 5 IFIMUP- Instituto de Física de Materiais Avançados, Nanotecnologia e Fotónica, Departamento de Física e Astronomia, Faculdade de Ciências, Universidade do Porto, Rua do Campo Alegre, s/n, Porto, 4169-007 Portugal; 6 ICBAS - Instituto de Ciências Biomédicas Abel Salazar, Universidade do Porto, Rua de Jorge Viterbo Ferreira, Porto, 4050-313 Portugal

**Keywords:** 3D in vitro models, Controlled drug delivery, Nanoparticles, NIPAM, Thermoresponsive, Triple negative breast cancer

## Abstract

Controlled release of chemotherapeutic drugs is a key factor in cancer therapy. In this regard, temperature triggered release of drugs offers a precise control over the release within the tumour region, thus avoiding premature drug release and unspecific toxicity. In this work a novel polymeric N-isopropylacrylamide-based nanocarrier was developed to carry and release doxorubicin in response to the natural higher temperature of solid triple negative breast cancer tumours. The nanocarrier was further functionalized with folic acid moieties to improve the targeting of these cells. The obtained results show that the produced nanoparticles presented a thermoresponsive behaviour with a transition temperature of 42 °C. Moreover, a high drug loading was achieved (9.7%) followed by a sustained and temperature dependent drug release (55% at 37 °C and 86% at 40 °C). Regarding biosafety, the nanoparticles were cytocompatible up to a concentration of 5,000 µg mL^− 1^, non-haemolytic, non-thrombogenic and anti-fouling, showing good properties to be intravenously administered. When tested against triple negative breast cancer cells in 2D models, a concentration and time dependent cytotoxic effect was observed, where folic acid-functionalized nanoparticles resulted in a lowering of the IC_50_ value in 77%, when compared with non-functionalized nanoparticles. Finally, a 3D in vitro model of the tumour was optimized, with the objective of better mimicking an in vivo scenario, where the NPs presented an effective tumour cell reduction in a temperature dependent manner. In conclusion, this work was able to optimize a novel nanoparticle to improve doxorubicin delivery in a controlled fashion, with specific targeting capabilities towards cancer cells, even in 3D systems where drug penetration is usually limited.

**Supplementary Information:**

The online version contains supplementary material available at 10.1007/s13346-025-01930-9.

## Introduction

In cancer therapy, drug loaded nanoparticles bring several advantages when compared to free drug administration. Among several benefits, the main ones regard the lower side effects and higher therapeutic capacity, owing to the outstanding tumour targeting capabilities of nanoparticles [1]. Yet, controlling the drug release profile is still a challenge with several nanoformulations, being characterized by the nonspecific drug diffusion during circulation or failed drug release in the tumour region [2, 3]. To address these issues, stimuli-responsive nanoparticles, meaning nanoformulations that respond to either intrinsic (e.g. tumoral pH and temperature) or extrinsic (e.g. magnetic and electric fields) stimuli, add a new layer of control on how drugs are transported and released [4–6].

In particular, temperature-responsive systems are very interesting due to their versatility, since they can be designed to respond to both extrinsic (e.g. hyperthermia) and intrinsic (e.g. natural higher temperature of solid tumours) stimuli. Usually, due to their high metabolic profile and dense microenvironment, solid tumours present a higher temperature when compared with healthy tissue, around 2 to 3 °C higher [7]. With this slight change, it is possible to design nanoparticles that release their cargo in this temperature range, thus resulting in a more specific drug release in the tumour microenvironment and in a decreased premature drug release. Poly(N-isopropylacrylamide) (PNIPAM) is a thermoresponsive polymer that, in water, presents a lower critical solution temperature (LCST) behaviour and a transition temperature of 32 °C [8, 9]. This polymer exhibits a contracted/coiled phase transition above the transition temperature, which is directly associated with the hydrogen bridges between the polymer side chains and water molecules, meaning that above 32 °C a phase separation occurs, turning the polymer less soluble in water [10]. Due to being below body temperature (~ 37 °C), the transition temperature of pure PNIPAM has no practical application for cancer therapy, yet NIPAM can be co-polymerised with hydrophilic monomers to increase this value, a process that allows a fine control over it by adjusting the ratio of each monomer in the final copolymer [11, 12]. By creating a more hydrophilic co-polymer, additional hydrogen bridges are formed with water molecules, thus more thermal energy is required for hydrophobic interactions between the polymer chains to occur, which increases the temperature transition of the co-polymer [6]. Note that, in addition to LCST, some polymers can also exhibit an upper critical solution temperature (UCST) behaviour, which can also be explored for drug delivery applications. With UCST, the polymers become miscible with a solvent above a specific temperature, contrary to LCST polymers which become immiscible [13]. With these polymers, above a specific temperature, the polymer solubilises, thus leading to the release of the entrapped drug [14].

Triple negative breast cancer (TNBC) is the most aggressive subtype of breast cancer for which there are no approved targeted therapies, being mainly treated by chemotherapy using anthracyclines such as doxorubicin (DOX). This drug acts mainly by inhibiting topoisomerase II and by DNA damage through free radical generation [15]. Yet, DOX has a non-specific activity, inducing extensive damage in healthy tissues, being commonly associated with irreversible cardiac toxicity and other side-effects such as skin sores, general pain, myelosuppression and even increased risk of infections and secondary tumour formation [16–18]. To try and overcome these side effects, liposomal doxorubicin (DOX-loaded PEGylated liposomes) has been introduced into the market and commercialized as Doxil^®^ [19]. However, this formulation does not present a stimuli-triggered drug release profile and does not have an active targeting mechanism, thus relying on the enhanced permeability and retention (EPR) effect. In fact, targeting TNBC is very challenging since it does not overexpress the commonly overexpressed breast cancer receptors (progesterone, estrogen and the HER2 protein), yet other receptors such as the folate receptor-α (FOLR1) can be explored [20–22]. FOLR1 expression in TNBC is not well defined, yet several studies report that this receptor is overexpressed in 35 to 68% of TNBC cases [23]. Clinically, this receptor is associated with a poor prognosis of the disease, yet targeting it has shown to be beneficial for an efficient drug delivery to TNBC cells [24]. For example, Sen et al., reported that folic acid functionalized peptide-based nanoparticles loaded with ormeloxifene, resulted in a significant increase in nanoparticle cellular internalization. This functionalization resulted in an improved cancer cell death by lowering the IC_50_ value from 7.3 to 3.5 µg mL^− 1^ with non-functionalized and functionalized nanoparticles, respectively [25].

Here, this study reports the production of a novel folic acid functionalized NIPAM based nanocarrier for the targeted and temperature-controlled transport/release of doxorubicin to treat TNBC. The nanoparticles were designed to be thermo-responsive, hydrophilic and easily functionalized. In their composition, the NIPAM monomer was introduced as the thermo-responsive moiety, while PEGMA was introduced to increase the hydrophilicity of the system and subsequently the transition temperature. Finally, AA was introduced to provide an exposed and highly reactive primary amine that could be used to functionalize the surface of the nanoparticles. In this work, folic acid was conjugated through the carbodiimide chemistry to form an amide bond between the γ-carboxyl group of folic acid and the free pendant amine in the polymer. This specific formulation provided the nanocarrier with a high transition temperature to avoid excessive premature drug release, to be both cytocompatible and hemocompatible and to increase cancer cell targeting through folate receptors, characteristics that are not fully explored in other works. Moreover, besides the common tests in 2D cultures, the efficacy of this formulation was also validated in 3D cultures, as they closely resemble in vivo conditions, thus improving the evaluation of the treatment efficacy and toxicity and providing an alternative to animal testing.

## Materials and methods

### Materials

N-isopropylacrylamide (NIPAM, 99%), OH-terminated Polyethyleneglycolmethacrylate (PEGMA, MW 360, 99%), allylamine (AA, 99%), N, N´-Methylenebisacrylamide (MBA, 99%), sodium dodecyl sulphate (SDS, 99%), ammonium persulfate (APS, 99%), 1-Ethyl-3-(3-dimethylaminopropyl)carbodiimide (EDC, 99%), N-hydroxysuccinimide (NHS, 99%), 2-(N-morpholino)ethanesulfonic acid (MES, 99%), dimethyl sulfoxide (DMSO, 99.9%) and doxorubicin hydrochloride (DOX, 99.99%) were all acquired from Merck, USA. Folic acid (FA, 99.9%) was acquired from ThermoFisher Scientific, USA. All reagents were used as obtained, without any purification step. Ultrapure water (resistivity 18.2 MΩ cm) was used at all times unless when specified.

### Nanoparticles synthesis

Bare thermo-responsive nanoparticles (BNPs) were produced by the free radical polymerization in water of the NIPAM, PEGMA and AA monomers, using MBA as the crosslinker, SDS as the surfactant and APS as the initiator. In more detail, in a 100 mL three-neck round bottom flask, 49 mL of ultrapure water were mixed with the monomers, crosslinker and surfactant, to make a homogeneous solution containing 325 mg of NIPAM, 13.1 mg of MBA, 80 µL of a SDS solution (20% w/v), 30 µL of AA and 25 µL of PEGMA. After solubilization, the solution was placed in an oil bath at 60 °C and purged, for 30 min, with N_2_ to generate an inert atmosphere. In parallel, 28.5 mg of APS were dissolved in 1 mL of ultrapure water and injected into the previous solution using a needle under N_2_ protection. The reaction proceeded at 60 °C under slow magnetic stirring (200 rpm). After 4 h, the nanoparticle suspension was allowed to naturally cool to room temperature and was dialyzed (SnakeSkin™ Dialysis Tubing 3.500 MW, Thermo Scientific, USA) against distilled water for 5 days with daily water exchanges. The BNPs were stored in water at room temperature for further use.

### Folic acid functionalization

Folic acid conjugation was performed by coupling FA with the free primary amines in the BNPs through the EDC/NHS carbodiimide chemistry. In detail, 107 mg of MES were dissolved in 11 mL of the BNPs suspension and the pH was adjusted to 5.4 ± 0.2 with 1 M NaOH. In parallel, 1 mg of FA, 10 mg of EDC and 10 mg of NHS were dissolved in 1 mL of DMSO and reacted for 1 h to activate the carboxyl groups of FA. Then, 500 µL of the activated FA solution were added dropwise to the BNPs suspension and stirred at 200 rpm for 24 h. Finally, the functionalized nanoparticles (FANPs) were dialyzed as described in the “Nanoparticles synthesis” section, lyophilized at -80 °C for 24 h and stored at 4 °C.

### Doxorubicin loading and release

For the loading of DOX into the nanoparticles, a DOX solution (0.6 mg mL^− 1^) in PBS (pH 7.4) was prepared. After dissolving the drug, lyophilized BNPs and FANPs were dispersed to a concentration of 5 mg mL^− 1^. The suspensions were then stirred (200 rpm) overnight at room temperature. Unloaded DOX was isolated by centrifugation (14000 rpm for 30 min), the supernatant filtered through a 200 nm syringe filter and the absorbance (λ = 490 nm) was measured using a Synergy MX fluorimeter (BioTek, USA). To determine the concentration, a DOX calibration curve was prepared (R^2^ = 0.999). Finally, encapsulation efficiency (EE) and drug loading capacity (LC) percentages were determined according to Eqs. 1 and 2, respectively, where Mi represents the initial mass of DOX, Mn represents the mass of non-encapsulated DOX and Mµ represents the initial mass of NPs. DOX loaded nanoparticles (BNPs-D and FANPs-D) were lyophilized (-80 °C) for 24 h in the presence of dextran (5% solution) as a cryoprotectant and stored, protected from light, at 4 °C for further use.

1 $$\:EE\left(\%\right)=\frac{\left(Mi-Mn\right)}{Mi}\times\:100$$

2 $$\:LC\left(\%\right)=\frac{(Mi-Mn)}{M\mu\:}\times\:100$$

For the drug release studies, lyophilized drug loaded nanoparticles were dispersed in 0.5 mL of PBS (pH 7.4) and placed inside a dialysis bag (SnakeSkin™ Dialysis Tubing 3.500 MW, Thermo Scientific, USA). Sink conditions were maintained by controlling the dialysate volume (minimum 10x times higher than the NPs volume). The bag was then placed in 15 mL of PBS (pH 7.4) at 37 and 40 °C. After each time point, 1 mL of the dialysate was collected and replaced with fresh PBS. Fluorescence (ex. 480 nm and em. 590 nm) was measured using a Synergy MX fluorimeter (BioTek, USA). A calibration curve (R^2^ = 0.990) was used to quantify the drug release.

### Physicochemical characterization

#### Morphology, size distribution, zeta-potential and thermoresponsiveness

Morphology was assessed by transmission electron microscopy (TEM) in a JEOL JEM-1400 Electron Microscope (JEOL Ltd., Tokyo, Japan) operating at an accelerating voltage of 120 kV. Images were obtained using a CCD digital camera Orious 1100 W (Gatan, USA). Particle size, size distribution and zeta potential measurements were determined through dynamic light scattering (DLS) and electrophoretic mobility in a Zetasizer Nano ZS (Malvern Panalytical, UK) with a scattering angle of 173° and a He-Ne 630 nm wavelength laser. To evaluate the thermoresponsive properties, size was measured at different temperatures through DLS. Temperature was increased in 5 °C steps from 25 to 50 °C with 5 min intervals in between steps for temperature stabilization purposes. Transition temperature values were determined by calculating the inflexion point of the obtained curves. Samples were prepared by dispersing the nanoparticles in ultrapure water and placing them in folded capillary cells (DTS1070, Malvern Panalytical, UK). Each sample was analysed 3 times and each experiment was repeated 3 times (*n* = 3).

#### Chemical analysis

Fourier-transform Infrared (FTIR) spectroscopy was employed to identify the chemical groups of each sample. For this, attenuated reflectance mode was used (FTIR-ATR), where 20 µL of a nanoparticle suspension (5 mg mL^− 1^) in ultrapure water were placed on top of the quartz crystal and allowed to fully dry. Spectra were recorded in a FTIR spectrometer (Perkin-Elmer, USA), with a 4 cm^− 1^ resolution, with an averaging of 32 scans per sample and within the 4000–400 cm^− 1^ spectral range.

Micro-Raman spectra were recorded using a Renishaw InVia Qontor spectrometer (Renishaw, UK), equipped with an optical microscope. This study was performed using a 1064 nm laser as excitation. The maximum laser power, on the sample surface, was 100 mW. The microscope is equipped with a 50x long working distance lens and the scattered light was registered in the 3500–100 cm^− 1^ spectral range. The spectral resolution is better than 2 cm^− 1^. For this analysis, lyophilized nanoparticles were placed on top of a glass substrate.

UV-Vis spectra were recorded using a UV-Vis spectrometer (Perkin Elmer, USA). For this, the nanoparticles were dispersed in ultrapure water and placed in a quartz cuvette. Scans were recorded in the spectral range of 200 to 600 nm.

Proton nuclear magnetic resonance (^1^H NMR) measurements were performed with a BRUKER AVANCE III 400 MHz (Bruker Corporation, USA) spectrometer at 25 °C in D_2_O. Chemical shifts are reported in ppm (δ units) and were referenced to the residual solvent signal.

### Biocompatibility studies

#### Cytocompatibility

L929 murine fibroblasts (CCL-1, ATCC) were cultured in α-MEM medium, supplemented with 10% FBS and 1% Pen/Strep, in a humidified atmosphere and incubated at 37 °C and 5% CO_2_. For cell viability studies, 1 × 10^4^ cells were seeded onto 96-well plates (working volume of 100 µL) and left to adhere for 24 h. BNPs and FANPs were added to the seeded cells at several concentrations in complete medium and incubated for 24, 48 and 72 h. After the time points, medium was removed and replaced with new complete medium containing 10% resazurin (stock solution of 0.1 mg mL^− 1^) and incubated for 3 h. Finally, the resazurin medium was transferred to 96-well black plates and the fluorescence (ex. 530 nm and em. 590 nm) was measured in a Synergy MX (Biotek, USA). Viability was normalized against the control group and defined as a percentage. Each experiment contained 5 replicates and was repeated 3 times (*n* = 3). The same experiment was repeated with MDA-MB-468 (HTB-132, ATCC) triple negative breast cancer cells cultured in DMEM medium supplemented with 10% FBS and 1% Pen/Strep, following the same protocol as described above.

#### Protein adsorption and hemocompatibility

To determine protein adsorption, 1 mg of nanoparticles was incubated with 1 mL of bovine serum albumin solution in PBS (pH 7.4) (BSA, 1 mg mL^− 1^) for 24 h at 37 °C. After the incubation period, the samples were centrifuged and the supernatant was collected. To determine the BSA concentration a Bradford method was employed by adding 150 µL of Coomassie reagent to 10 µL of sample, mixed for 30 s and allowed to incubate for 5 min at room temperature. Absorbance was read at 595 nm in a multiplate reader (Synergy MX, USA). A BSA standard curve was used to calculate BSA concentrations (R^2^ = 0.996).

Hemocompatibility was evaluated by the haemolytic and thrombogenic profile of the nanoparticles, under an ethics protocol (Ref. 90/19). For the haemolysis assay, red blood cells (RBCs) were extracted from human buffy coats by centrifugation at 400 g for 30 min. Following centrifugation, the upper layer was discarded and the pellet was washed 3x with PBS (pH 7.4). The nanoparticles were incubated with isolated RBCs (2 × 10^8^ cells mL^− 1^) in 96-well suspension plates for 3 h at 37 °C. Subsequently, the plates were centrifuged at 4000 rpm for 15 min. 100 µL of the supernatant were transferred to 96-well plates and the absorbance was measured at 380, 415 and 450 nm, using a microplate reader spectrophotometer Synergy MX (Biotek, USA). The amount of released haemoglobin (RH) was calculated following Eq. 3 where A415, A380, and A450 represent the absorbance values of the samples at 415 nm, 380 nm, and 450 nm, respectively. E is the molar absorptivity of oxyhaemoglobin at 415 nm. Hemolysis was calculated according to Eq. 4, where RH represents the total release of haemoglobin in Triton X-100 1%. The test was performed 3 individual times (*n* = 3) with 5 replicates within each assay.

3 $$\:RH\left(mg\:{dL}^{-1}\right)=\frac{(2\times\:{A}_{415}-({A}_{380}+{A}_{450})\times\:1000}{E}$$

4 $$\:Haemolysis\left(\%\right)=\frac{RHsample}{RHtotal}$$

The effect of the nanoparticles on plasma coagulation was evaluated by the plasma coagulation kinetics assay, using human plasma under an ethics protocol (Ref. 90/19). For this, silica nanoparticles and PBS were used as positive and negative controls, respectively. 50 µL of a nanoparticle suspension (4 mg mL^− 1^), or the controls, were mixed with 450 µL of human plasma and incubated for 30 min at 37 °C. After incubation, 50 µL of the nanoparticle + plasma mixture were placed in 96-well suspension plates, to which 50 µL of a CaCl_2_ solution (5 mM) were added. The absorbance was measured at 405 nm for 180 min, with 1 measurement per minute. The coagulation time was determined at the inflexion point of the obtained curves.

### Anti-cancer activity in 2D models

#### Cancer cell viability studies

MDA-MB-468 (HTB-132, ATCC) triple negative breast cancer cells were cultured in DMEM medium containing 10% FBS and 1% Pen/Strep, in a humidified incubator at 37 °C and 5% CO_2_. For cell viability studies, 1 × 10^4^ cells were seeded in 96-well plates (working volume of 100 µL) and allowed to adhere for 24 h. BNPs-D and FANPs-D were added to the seeded cells at several concentrations in complete medium and incubated for 24, 48 and 72 h. After the time points, the resazurin method was employed as in the “Cytocompatibility” section. Viability was normalized against the control group and defined as a percentage. Each experiment contained 5 replicates and was repeated 3 times (*n* = 3).

#### Folate receptor competition assay

MDA-MB-468 (HTB-132, ATCC) were grown and seeded in 96-well plates as in the “Cancer cell viability studies” section. Before the addition of the nanoparticles, some cell conditions were incubated with a FA solution (5.0 mM) in complete medium for 1 h to saturate the FOLR1 receptors. After incubation, BNPs-D and FANPs-D were added at a DOX concentration of 100 µM and incubated for 24 and 72 h. Finally, the resazurin method was employed as in the “Cytocompatibility” section. Viability was normalized against the control group and defined as a percentage. Each experiment contained 5 replicates and was repeated 3 times (*n* = 3).

### Anti-cancer activity in 3D models

#### Spheroids optimization

3D cell constructs (spheroids) were developed using commercially available micromolds (3D Petri Dish^®^, MicroTissues, USA), following the manufacturer’s instructions. Culture was performed in low-adhesion 12-well plates and the working volume was set to 1.5 mL. MDA-MB-468 cells were cultured as in the “Cancer cell viability studies” section, trypsinized and seeded in the agarose micromolds at different cell densities per spheroid (1 × 10^3^, 2.5 × 10^3^ and 5 × 10^3^). Medium was replaced every 2 days. At different timepoints (1, 4 and 7 days), cells were imaged by brightfield microscopy (ZOE™ Fluorescent Cell Imager, Bio-Rad Laboratories, USA). Size was evaluated through the ImageJ software version 1.8.0 (NIH, USA), by taking 4 diameter measurements in 3 randomly selected spheroids. Viability was assessed by the resazurin assay by replacing the growing medium with fresh medium containing 20% resazurin, followed by a 2 h incubation period. Medium was transferred to black 96-well plates and the signal fluorescence was measured (ex.530 nm, em.590 nm) in a plate reader (SynergyMX, BioTek, USA). To complement the viability studies and understand the structure of the spheroid, a live-dead assay was performed. After the timepoints, each mold was washed with PBS and incubated with Calcein-AM (1:1000 dilution) for 15 min in complete medium, followed by incubation with a solution of propidium iodide in PBS (25 µg mL^− 1^) for 15 min. Finally, the molds were washed with PBS and analysed by fluorescence (Calcein: λ = 488 nm; Propidium iodide: λ = 561 nm) microscopy using a SP5 confocal microscope (Leica Microsystems, Germany) and image analysis was performed with the LAS AF Lite software (Leica Microsystems, Germany) and with the ImageJ software version 1.8.0 (NIH, USA). Images were created by combining z-stacks.

#### NPs anti-cancer activity in 3D cultures

MDA-MB-468 spheroids were produced as described in the “Spheroids optimization” section. At day 4, medium was replaced with medium containing free drug or FANPs-D at several concentrations for 72 h at 37 and 40 °C. After 72 h, the spheroids were washed with PBS and incubated for 2 h with complete medium containing 20% resazurin. Medium was transferred to black 96-well plates and the signal fluorescence was measured (ex. 530 nm, em. 590 nm) in a plate reader (SynergyMX, BioTek, USA). Each experiment contained 4 replicates and was repeated 3 times (*n* = 3).

#### Doxorubicin penetration evaluation

Spheroids with an initial cell seeding density of 2.5 × 10^3^ cells per spheroid were produced as presented in the “Spheroids optimization” section. At day 4 of culture, the spheroids were exposed to the free drug or to FANPs-D at 37 and 40 °C. After 6 h, spheroids were washed 3x with PBS and fixed for 15 min with a 4% paraformaldehyde solution. After fixation, the molds were washed with PBS and analysed by fluorescence microscopy, taking advantage of DOX fluorescence (ex. 480 nm and em. 590 nm), using a SP5 confocal microscope (Leica Microsystems, Germany) and image analysis was performed with the LAS AF Lite software (Leica Microsystems, Germany) and with the ImageJ software version 1.8.0 (NIH, USA). Images were created by combining z-stacks.

### Ethics statement

Blood components were obtained from surplus buffy coats from healthy blood donors, kindly provided by the Immunohemotherapy Department of Centro Hospitalar Universitário São João (CHUSJ), Porto, Portugal. Procedures were approved by the Centro Hospitalar Universitário São João Ethics Committee (protocol 90/19). Blood donors provided informed written consent that the by-products of their blood collections could be used for research purposes.

### Statistical analysis

Statistical analysis was performed with the GraphPad Prism 8 software (GraphPad, USA). Data are represented as mean ± standard deviation (SD). Statistical analysis was performed using the analysis of variance One-way ANOVA or Two-way ANOVA, followed by Tukey’s multiple comparison test. Statistical significance was considered when *p* < 0.05 and represented with * for *p* < 0.05, ** for *p* < 0.01, *** for *p* < 0.001 and **** for *p* < 0.0001.

## Results and discussion

### Size, charge and thermoresponsive behaviour

**Fig. 1:**
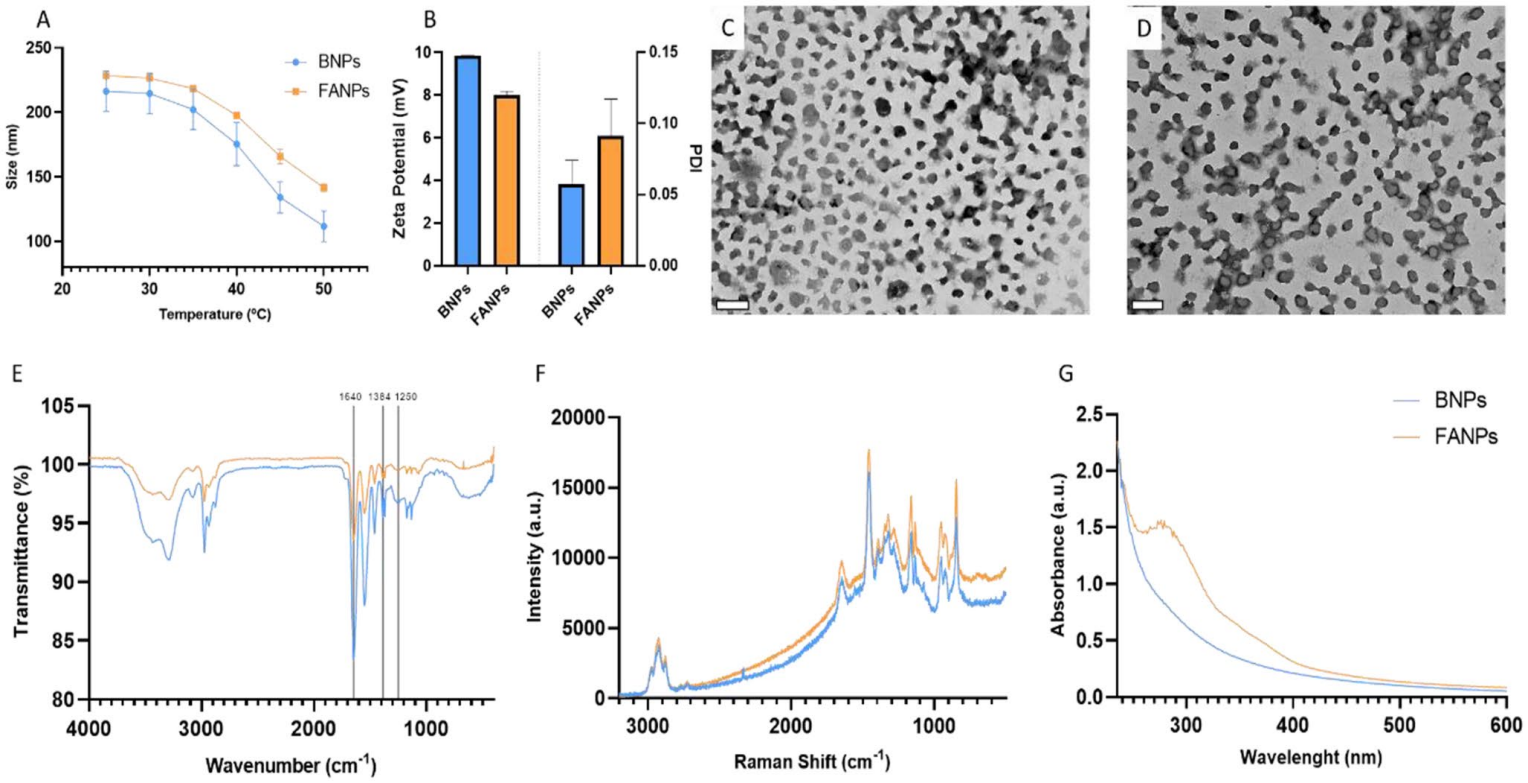
Physicochemical characterization. (**A**) Size variation, in water, of BNPs and FANPs through DLS between a temperature range of 25 to 50 °C. (**B**) Zeta-potential and polydispersity index (PDi) measurements through DLS, in water, at 25 °C. (**C**) TEM image of BNPs in their dry state. Scale bar: 500 nm. (**D**) TEM image of FANPs in their dry state. Scale bar: 500 nm. (**E**) ATR-FTIR spectra of BNPS and FANPs. (**F**) RAMAN spectra of lyophilized BNPS and FANPs. (**G**) UV-Vis spectra of BNPs and FANPs revealing the characteristic absorption peaks of folic acid

Both BNPs and FANPs presented a thermoresponsive behaviour (Fig. 1A), indicating that the particle size reduced as a function of temperature increase, from 216 to 111 nm and 228 to 142 nm, corresponding to a 49% and 37% contraction, respectively. The size of the produced nanoparticles was mainly controlled by the presence of SDS as a surfactant that limited the polymer growth. Regarding the surface charge (Fig. 1B), BNPs presented a positive zeta potential of 9.89 mV due to the presence of the primary amine in the AA monomer, while in FANPs the zeta potential reduced to 8.86 mV due to the more negative nature of this ligand [26]. Moreover, both samples presented a uniform size distribution, as shown by the low polydispersity index (PDI < 0.2) (Fig. 1B). Regarding the transition temperature, obtained from the DLS analysis, BNPs and FANPs presented values of 41.8 and 42.3 °C, respectively. As mentioned in The Introduction, the transition temperature of pure PNIPAM is 32 °C in water, but due to the presence of the PEGMA and AA hydrophilic monomers in the final nanocarrier, the value increased thus allowing the application of these nanoparticles in solid tumour therapy [9, 12]. It is important to note that, despite not being statistically significant (*p* > 0.05), the functionalization of FA on the BNPs increased the transition temperature value by 0.5 °C, likely due to its hydrophilic nature. This effect was also observed with other hydrophilic biomolecules such as DNA and antibodies [27, 28].

### Physicochemical characterization

Transmission electron microscopy (TEM) images (Fig. 1C and D) confirmed the synthesis of polymeric nanoparticles with a homogeneous size distribution (~ 200 nm) and a spherical morphology. During this analysis, no significant differences were observed in terms of size when comparing BNPs with FANPs.

From the FTIR spectra (Fig. 1E), both spectra comprise peaks at 3500 cm^− 1^ corresponding to N-H stretching, ~ 1640 cm^− 1^ corresponding to C = O stretching, relative to NIPAM. The peak at 1548 cm^− 1^ corresponds to C-N stretching, relative to NIPAM and AA [29, 30]. The peak at ~ 3299 cm^− 1^ corresponds to the O-H stretching of water. The -C-H stretching region is located between 2975 cm^− 1^ and 2875 cm^− 1^ and is represented by three peaks. Similarly, the C–H deformation region is also characterized by three peaks at 1460 cm^− 1^, 1384 cm^− 1^ and 1371 cm^− 1^ [29]. Peaks associated with C-O-C, C = O and C-O can be observed at 1250 cm^− 1^, 1718 cm^− 1^ and 1106 cm^− 1^, respectively that are attributed to the presence of PEGMA in the polymer [31]. In the RAMAN spectra (Fig. 1F), most of the vibrational bands are in the range from 500 to 3000 cm^− 1^. The peak at 840 cm^− 1^ is associated with C-C stretching in the monomers, while the ones at 920 and 950 cm^− 1^ correspond to C-C stretching of the polymer backbone. Peaks associated with CH_3_ rocking, C-O-C stretching, C-H bending and CH_3_ symmetric deformation can be observed at 1125, 1165, 1450 and 1390 cm^− 1^, respectively. C-N stretching can be found in peaks at 1640 cm^− 1^, due to the presence of NIPAM and AA. The CH_3_ antisymmetric stretching, CH_2_ symmetric stretching and CH_3_ symmetric stretching vibrations are found at 2970, 2920 and 2870 cm^− 1^, respectively [32]. Finally, a band associated with C = C bonds found in folic acid were detected between 680 and 700 cm^− 1^ [33]. For a more straightforward interpretation, a summary of these results may be found in Tables S1 and S2. ^1^H NMR (Fig. S1) exhibits peaks of protons of the isopropyl group of NIPAM (δ = 1.17 and 3.91 ppm) [34]. Protons of PEGMA side chains (O-CH_2_-CH_2_-O) presented as a sharp peak at δ = 3.72 ppm, while protons of the polymer backbone appear at δ = 2.16, 2.04, 1.74, 1.62 and 1.48 ppm [34, 35]. Allyl amine associated protons can also be faintly observed at δ = 2.81 ppm [36]. Folic acid was also present however, due to the low concentration of this molecule in the polymer, only faint peaks were detected (δ = 7.75, 7.00, 4.27 and 4.26 ppm) [37]. Finally, UV-Vis spectra (Fig. 1G) of BNPs, did not show any absorptions bands in the spectral range of 200 to 600 nm. Yet, FANPs, presented the characteristics peaks of folic acid at 282 and 365 nm, thus confirming the presence of this molecule in the nanoparticles [38].

### Drug loading and release

**Fig. 2:**
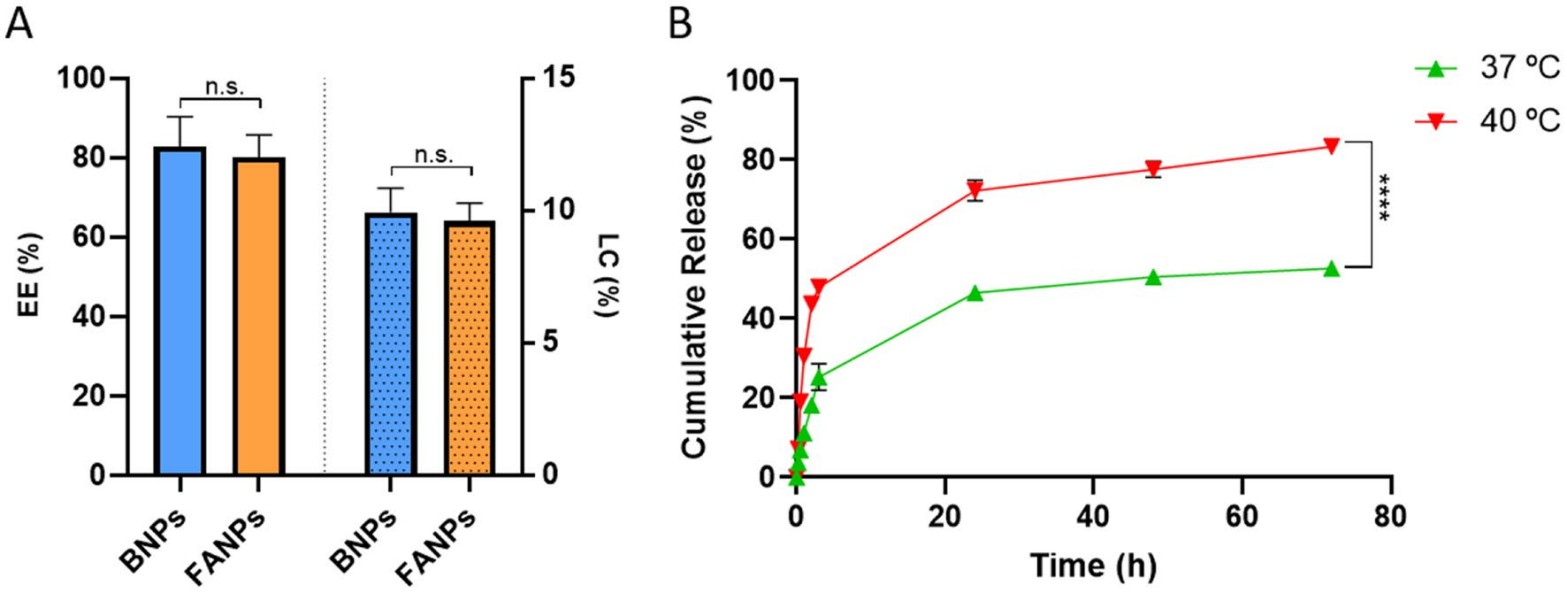
Doxorubicin loading and release. (**A**) Drug loading efficiency and loading capacity of BNPs and FANPs. (**B**) Drug release profile of FANPs, in PBS, at 37 and 40 °C

Drug loading was studied in terms of encapsulation efficiency (EE) and loading capacity (LC). EE refers to the efficiency of the encapsulation method, while LC shows the amount of drug that the nanoparticles can hold. Both BNPs and FANPs were able to incorporate DOX (Fig. 2A) within the polymer matrix with a mean encapsulation efficiency of 83% and 80%, respectively. Regarding loading capacity, the systems achieved a high mean loading content of 9.9% and 9.7%, respectively. These sets of values are comparable or higher than reported in literature for similar DOX nanocarriers [39–41]. Moreover, the presence of FA did not significantly influence the drug loading process (*p* > 0.05). The drug loading mechanism of DOX into these polymer constructs is supposed to be due to hydrogen bridges between the drug molecule and the monomer subunits of the co-polymer and by electrostatic interactions as explained by Kwon et al. [42]. Regarding the drug release profile (Fig. 2B), a sustained release was observed over 72 h, achieving a 55% and 86% release at 37 and 40 °C, respectively for FANPs. When comparing both curves, a significant difference was detected in the release profile (f_2_ = 35). In comparison with BNPs (Fig. S2), a similar release profile was observed (f_2_ > 95). Yar et al., developed NIPAM-based nanoparticles with a transition temperature of 38 °C and also verified a similar release profile. When tested at 42 °C, a significantly higher doxorubicin release was achieved compared to 37 °C [43]. In more detail, for FANPs, an initial DOX release occurred in 3 h (31% at 37 °C vs. 54% at 40 °C), followed by a sustained release pattern. When fitted against different drug release kinetic models (Table 1), the Korsmeyer-Peppas model presented the best correlation coefficient (R^2^ = 0.94) being associated with a Fickian release at both temperatures (*n* < 0.45). These results may indicate that diffusion governs the drug release mechanism. It is also important to note that the diffusional exponent (n) decreases at 40 °C which correlates well with the expected drug release mechanism. At higher temperatures, hydrophobic interactions dominate inside the polymer matrix, thus increasing the diffusion of the drug into the outer water rich environment.

**Table 1:** Fitting of the FANPs drug release kinetic models

Temperature	Zero-order		First-order			Higuchi		Korsmeyer-Peppas		
	*R* ^2^	k_0_	*R* ^2^	Q_t_	k	*R* ^2^	kH	*R* ^2^	k	*n*
37 °C	0.4783	0.9342	0.9381	50.45	0.248	0.8580	7.47	0.9482	14.45	0.324
40 °C	0.1109	0.1109	0.9392	52.30	0.312	0.6737	11.96	0.9438	30.28	0.251

### Cytocompatibility

**Fig. 3:**
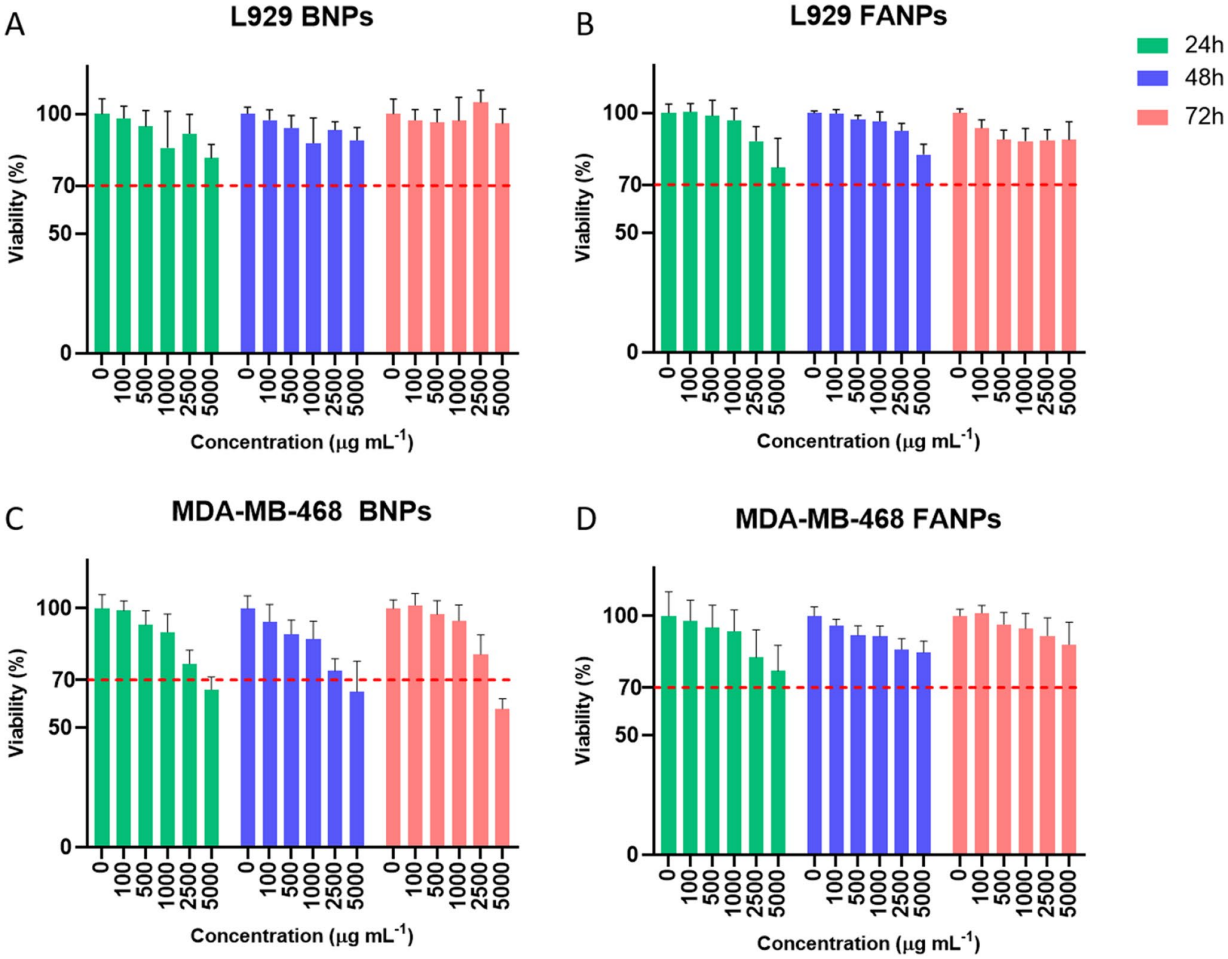
Nanoparticles cytocompatibility when tested against healthy fibroblasts (L929 cell line) and triple negative breast cancer cells (MDA-MB-468 cell line). Cells were exposed to the BNPs and FANPs, without drug, in a range from 0 to 5000 µg mL^− 1^ over periods of 24, 48 and 72 h. A red dashed line at 70% indicates the viability threshold

Cytocompatibility of the nanoparticles without drug was evaluated in both healthy fibroblast cells and TNBC cells up to a concentration of 5000 µg mL^− 1^ and up to 72 h of exposure (Fig. 3). BNPs proved to be cytotoxic (viability below 70%, according to ISO 10993-5) towards TNBC cells above 5000 µg mL^− 1^. Contrarily, post FA functionalization, none of the tested concentrations were toxic towards the tested cell lines. Such differences were not expected, yet several factors could have contributed to the lesser toxicity of FANPs namely, lower surface charge and higher hydrophilicity. Yet, it is important to note that BNPs only presented toxic effects at concentrations above 5000 µg mL^− 1^, that are not required for an effective therapy due to their high drug loading capacity.

### Blood compatibility and protein adsorption

**Fig. 4:**
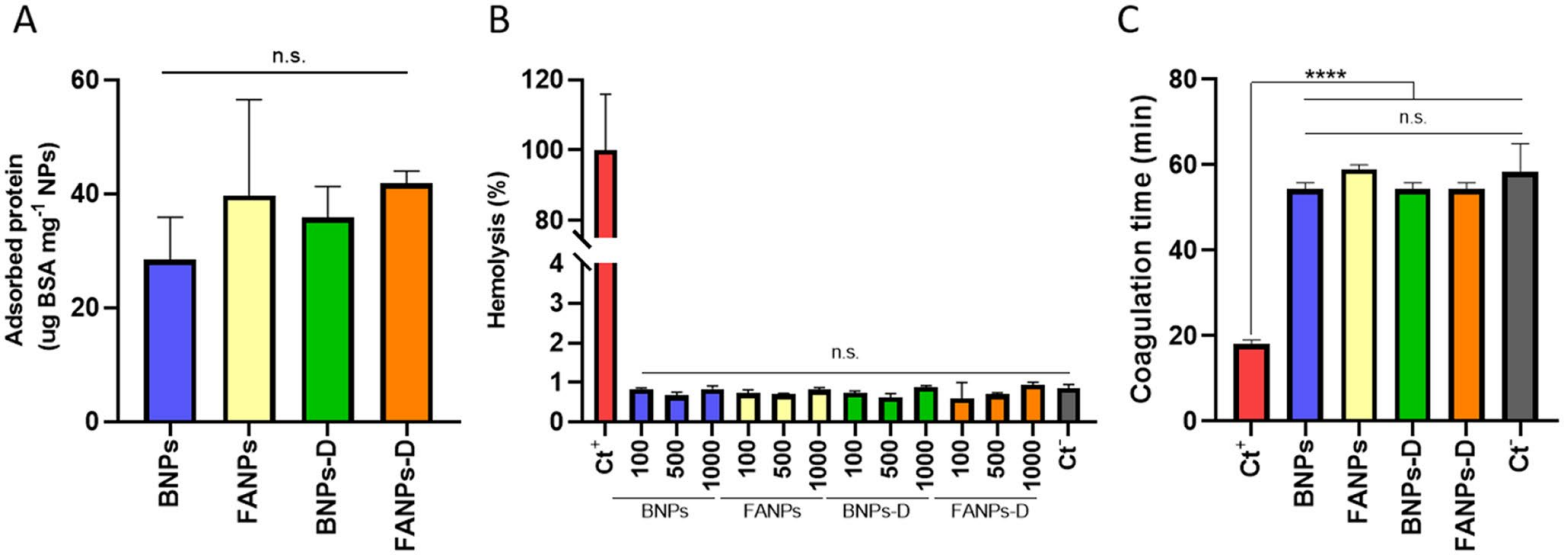
Protein adsorption and hemocompatibility. (**A**) Albumin adsorption after 24 h of contact at 37 °C, detected by the Bradford method. (**B**) Haemolysis rate of BNPs, FANPs and their respective drug loaded versions (BNPs-D and FANPs-D). Ct^−^: PBS; Ct^+^: Triton-X (1% in PBS). (**C**) Plasma coagulation time of the nanoparticles in contact with human platelet-poor plasma. Ct^−^: PBS; Ct^+^: SiO_2_ nanoparticles

Intravenous systems should be inert in contact with blood to achieve a safe and effective administration. Moreover, protein adsorption should be minimized to avoid recognition by the immune system and increase the circulation time of the nanoparticles, thus achieving a higher tumour accumulation [44]. Firstly, protein adsorption was evaluated using BSA since it is the most abundant protein in the blood tissue. The results, Fig. 4A, show that all the formulations only adsorbed below 40 µg of BSA per mg of nanoparticles (*p* > 0.05), corresponding to an adsorption below 4%. Protein adsorption is highly influenced by the surface properties, where hydrophilicity plays a major role. Generally, hydrophilic surfaces create a hydration layer that protects the surface from unspecific protein adsorption [45, 46]. In NIPAM-based systems, it has been described that protein adsorption is highly influenced by temperature, where temperatures above the transition temperature of the nanoparticles increase protein adsorption due to the increased hydrophobicity of the nanoparticles [47]. Yet, in a study by Prawatborisut et al., the authors showed that thermoresponsive systems are suitable to promote the specific adsorption of apolipoproteins, instead of albumin. These proteins contribute to the stealth effect during systemic administrations in in vivo systems, thus increasing the circulation time of the nanoparticles, while retaining cell targeting capabilities [48]. It is also important to note that surface decorations, such as folic acid, may also change the properties of the nanoparticles depending on the functionalization density. Such alterations may influence the protein adsorption mechanism, ultimately affecting the in vivo performance of the formulations. This makes protein adsorption studies important during nanoparticle development and should be conducted in a case-by-case fashion.

Compatibility with red blood cells was assessed by the haemolysis assay and as shown in Fig. 4B, all the different NPs are non-haemolytic (haemolysis < 5%) [49]. This showed that the NPs were not negatively interacting with erythrocytes which can be explained by the close to neutral surface charge and their hydrophilic nature [50]. Besides erythrocytes, the NPs did not promote the formation of blood clots, which could be life-threatening if formed during treatment. Based on the plasma coagulation assay, Fig. 4C, none of the formulations promoted plasma coagulation and presented a very similar behaviour to the negative control (*p* > 0.05). During the clotting process, in the intrinsic pathway, thrombogenic nanomaterials activate the coagulation factor XII which then proceeds with the activation of several other factors in the coagulation cascade until a fibrin clot is formed [51]. In the case of the produced nanoparticles, induction of coagulation did not occur which can be explained by their hydrophilic nature and low protein adsorption, yet it has been reported that other aspects such as size, surface charge and morphology can influence this behaviour, meaning that the thrombogenic activity of nanoparticles should be analysed on a case-by-case basis [52, 53].

### 2D in vitro anti-cancer activity

**Fig. 5:**
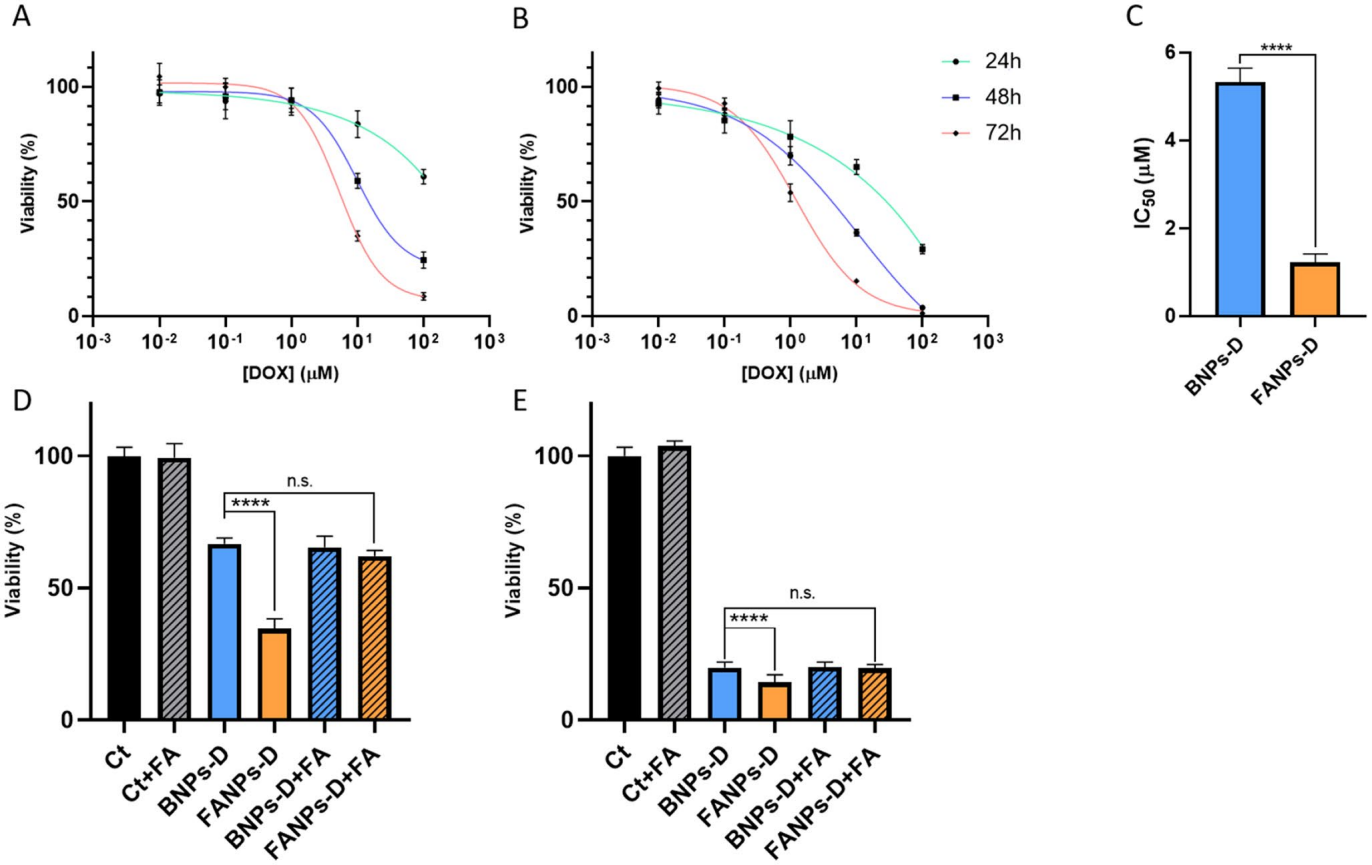
2D Anti-cancer activity studies of drug loaded BNPs and FANPs. (**A**) Viability of MDA-MB-468 cells when exposed to several doses of drug loaded BNPs over periods of 24, 48 and 72 h. (**B**) Viability of MDA-MB-468 cells when exposed to several doses of drug loaded FANPs over periods of 24, 48 and 72 h. (**C**) Calculated IC_50_ values of BNPs and FANPs after 72 h of exposure. (**D** and **E**) Competition assay after 24 and 72 h of exposure to the nanoparticles, respectively (+ FA indicates the groups with saturated FOLR1 receptors)

2D in vitro cell cultures are a standard practice in cytotoxicity evaluation. From Fig. 5A and B, a dose and time dependent cell viability decrease was observed when TNBC cells were exposed to BNPs-D and FANPs-D, respectively. After 72 h of exposure, the IC_50_ values were 5.33 µM and 1.22 µM for BNPs-D and FANPs-D, respectively (Fig. 5C). The functionalization of the BNPs with FA led to a 77% significant reduction of the IC_50_ value, indicating an active targeting mechanism. In a study by Kim et al., where folic acid functionalized doxorubicin loaded NIPAM-based nanoparticles were developed, the authors also observed a concentration and time dependent cell viability decrease. In the same study, the authors also showed that the empty nanoparticles were non-toxic, however they did not perform a dose-depended evaluation [54]. The competition assay (Fig. 5D and E) showed that when the folate receptors were saturated with FA and exposed to FANPs-D, a similar (*p* > 0.05) viability decrease was observed compared to BNPs-D. These results reveal that FA functionalization is a key parameter for a better therapeutic outcome and is expected to play an even more prominent role in vivo due to its targeting function.

### 3D spheroid characteristics

**Fig. 6:**
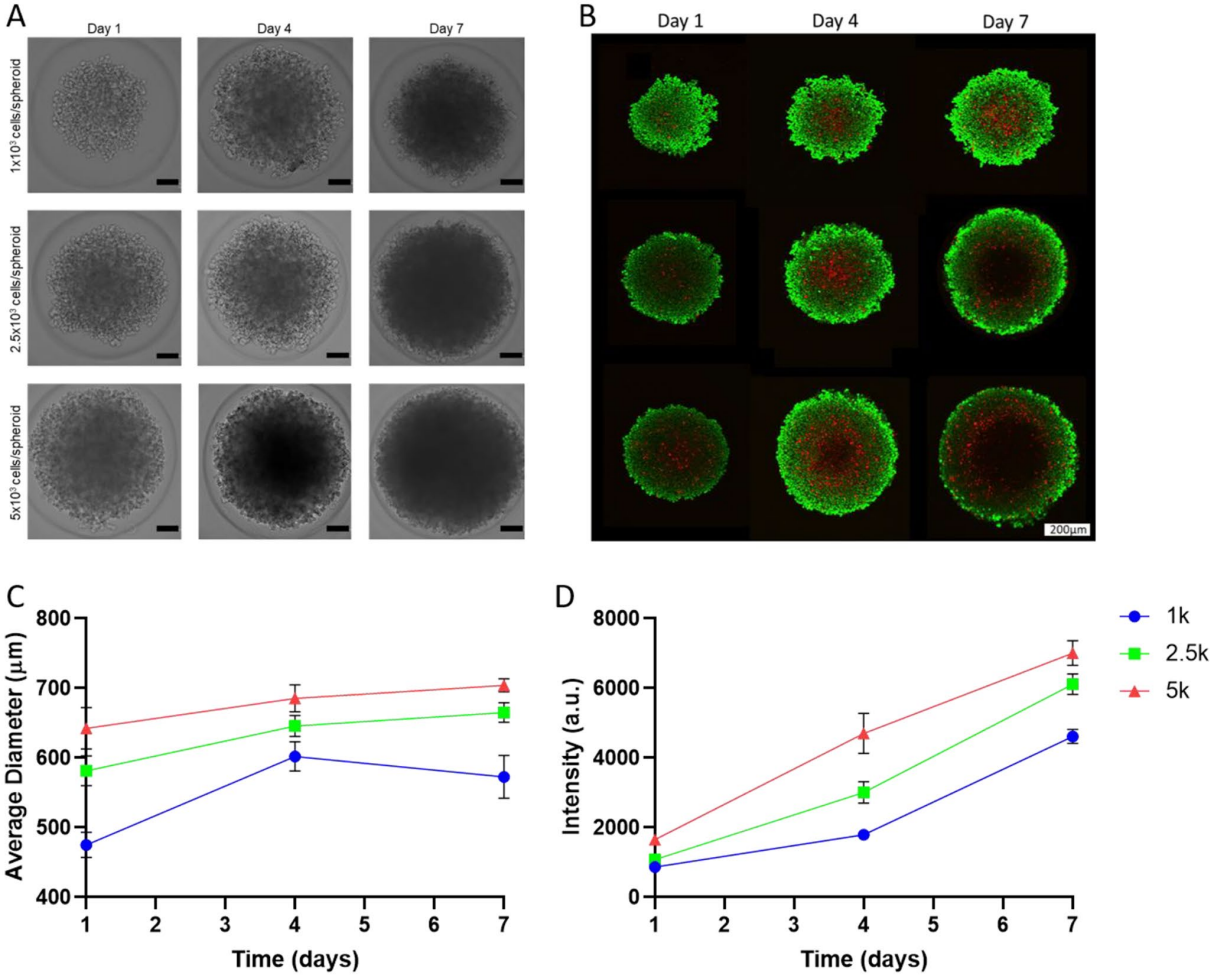
3D spheroid optimization using the MDA-MB-468 cell line. (**A**) Bright-field images of the spheroids with different cell seeding densities over a period of 7 days. Scale bar: 100 μm. (**B**) Confocal imaging of live-dead staining (red – dead cells; green – live cells). Scale bar: 200 μm. (**C**) Average diameter of the spheroids over a period of 7 days. (**D**) Spheroid metabolic activity over a period of 7 days

3D in vitro cell models are emerging as the new standard in cancer research. These models mimic the 3D structure of naturally occurring solid tumours, thus providing more accurate results prior to pre-clinical in vivo testing. The spheroids were produced in agarose molds that lower the cell-surface adhesion, thus promoting cell-cell interactions and ultimately result in the large-scale production of TNBC spheroids with excellent reproducibility in shape and size. Firstly, cell seeding density needed to be evaluated over time and for this, three different cell densities were chosen (1,000, 2,500 and 5,000 cells per spheroid). From the brightfield images (Fig. 6A) at day 1, loose cell aggregates can be observed. Over time, until day 7, the spheroids became more round and denser probably due to cell proliferation and increased extracellular matrix deposition [55]. In terms of size (Fig. 6C), all conditions continuously increased in size over time until saturating between 600 and 700 μm, while continuing metabolic active over time (Fig. 6D). Finally, from the live-dead assay (Fig. 6B), two different regions could be observed. The central core of the spheroids was mainly composed of dead cells as indicated by the red colour, while the outside limits were composed of live cells (green colour). As the spheroids grew over time, the dead core increased in size. Such structure is characteristic of naturally occurring tumours, where a necrotic core is found at the centre, being explained by the lack of oxygen (hypoxia) and nutrients that reach the dense core of the tumour, coated with an active and proliferating outer shell [56, 57].

All the conditions were able to maintain metabolically active cells up to 7 days and kept a stable diameter (around 600 μm), while still retaining a representative tumour structure. Therefore, the middle point (2,500 cells per spheroid) was chosen for further studies.

### 3D in vitro anti-cancer activity and drug penetration

3D anti-cancer activity studies of the free drug and drug loaded FANPs, were performed after the fourth day of maturation of the spheroids at 37 °C and 40 °C. Note that the controls were not influenced by the change in incubation temperature (Fig. S3).

**Fig. 7:**
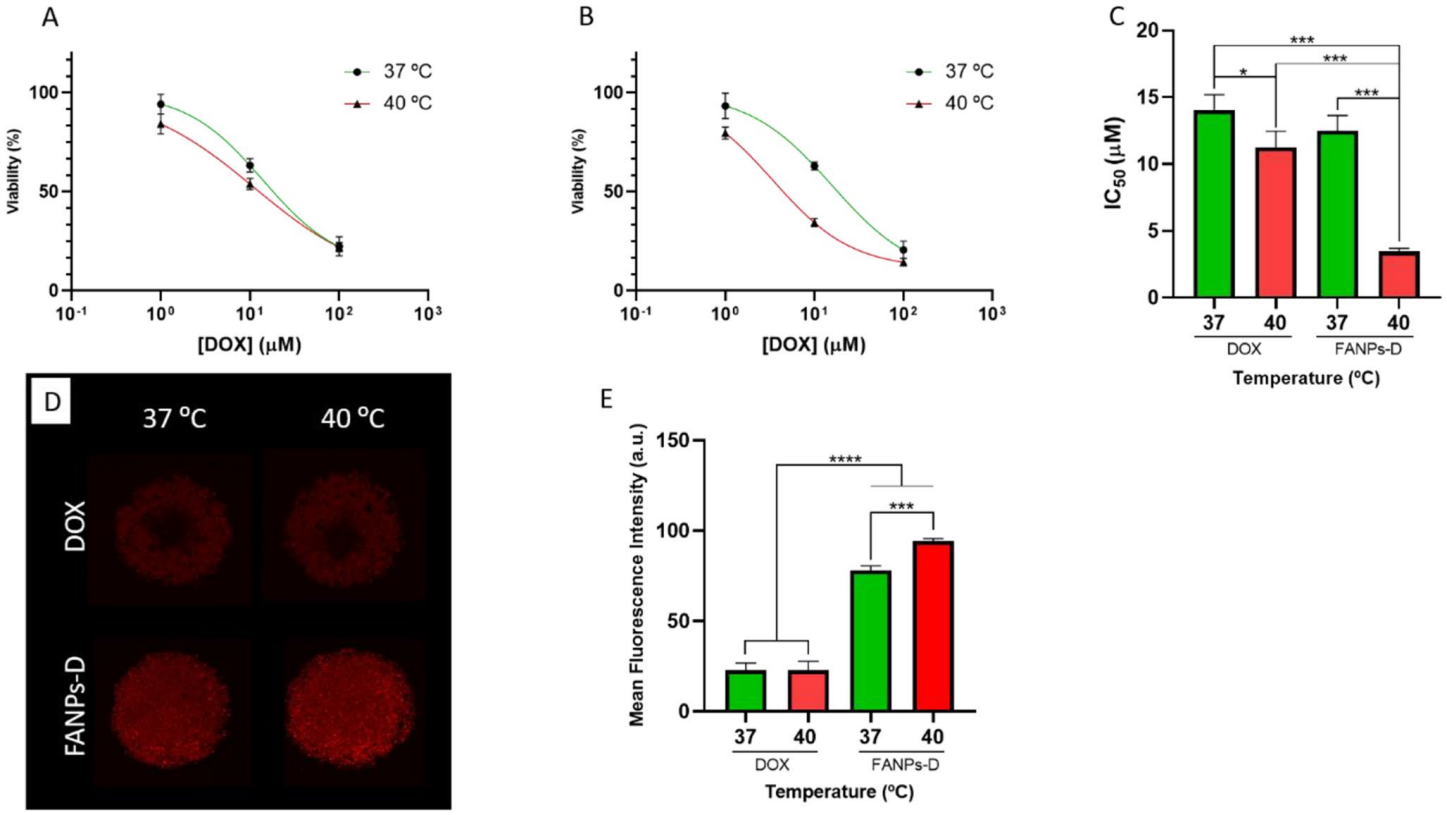
3D Anti-cancer activity studies of drug loaded FANPs. (**A**) Viability of MDA-MB-468 spheroids when exposed to several doses of free drug for 72 h at 37 °C (physiologic temperature) and 40 °C (tumoral temperature). (**B**) Viability of MDA-MB-468 spheroids when exposed to several doses of drug loaded FANPs for 72 h at 37 °C (physiologic temperature) and 40 °C (tumoral temperature). (**C**) Calculated IC_50_ values of free drug and drug loaded FANPs after 72 h of exposure at 37 and 40 °C. (**D**) Confocal microscopy of doxorubicin spheroid penetration at 37 and 40 °C. (**E**) Doxorubicin signal intensities in the spheroids at 37 and 40 °C

For both the free drug (Fig. 7A) and the drug loaded FANPs (Fig. 7B), a concentration dependent decrease in the spheroid’s viability was observed, which was expected due to the cytotoxic nature of the drug and the previous 2D assays. Interestingly, an IC_50_ significant decrease was achieved (Fig. 7C) for both the free drug and the FANPs when the temperature was increased to 40 °C. However, the FAPNs presented a significantly higher decrease of the IC_50_ value (3.46 µM). Such effect correlates well with the confocal images of the drug penetration (Fig. 7D). In these images, the free drug was not able to penetrate the entire volume of spheroid, and the temperature had no significant effect on the signal intensity (Fig. 7E). Contrarily, the drug loaded FANPs were able to penetrate the entirety of the spheroid (Fig. 7D) and retain the drug for a larger period of time. Similar findings were also reported by Lee et al., where DOX loaded nanoparticles achieved a higher penetration [58]. Such effect derives from different factors such as the complexes between the free drug and the dense extracellular matrix, which impairs drug diffusion, plus the fact that nanoparticles are transported between each cell through transcytosis, thus enabling a uniform distribution of the drug throughout the entire volume of the spheroid [59, 60]. Additionally, the temperature of 40 °C induced a significantly higher signal intensity (Fig. 7E), which can be explained by the drug release experiments.

## Conclusions

Targeting cancer cells and controlling drug release profiles are two major challenges in tumour chemotherapy. In this work, thermoresponsive nanoparticles with a high transition temperature (42 °C) and a stable, cytocompatible (up to 5 mg mL^− 1^), and hemocompatible profile were developed, demonstrating a targeted and improved control over the drug release profile of doxorubicin. In contrast to market-available products, such as Doxil^®^, this system was able to target TNBC cells in both 2D and 3D in vitro models, as well as to release doxorubicin in response to a specific endogenous stimulus (55% at 37 °C and 86% at 40 °C), rendering it an easy and effective strategy to induce tumour cell death. Currently, one limitation of these nanoparticles is their non-biodegradable profile which could be corrected by including a cleavable crosslinker instead of MBA. Moreover, folic acid could be easily exchanged by other targeting moieties such as antibodies, due to the easy functionalization chemistry of the nanoparticles, to target different receptors. Additionally, despite the promising confirmed properties in terms of biocompatibility and drug delivery, future in vivo studies are required to further validate the efficacy and safety profiles of this nanoformulation, mainly in terms of pharmacokinetics and tumour targeting. Finally, future developments could explore the incorporation of magnetic or laser responsive components as a promising strategy to achieve an externally activated release of drugs.

## Electronic supplementary material

Below is the link to the electronic supplementary material.

Supplementary Material 1

## Data Availability

Data will be made available upon request.
