# Supplementary material for: Thermoresponsive nanoparticles for targeted and controlled delivery of doxorubicin in triple negative breast cancer: a 2D and 3D in vitro evaluation
Source: Drug Deliv Transl Res. 2025 Jul 28;16(1):108–23. doi: 10.1007/s13346-025-01930-9 (PMC12682917; doi:10.1007/s13346-025-01930-9)
Supplement: Supplementary file 1 — Supplementary Material 1 [file 13346_2025_1930_MOESM1_ESM.docx]

Supplementary information for the article:

**Thermoresponsive Smart Nanoparticles for Targeted and Controlled Delivery of Doxorubicin in Triple Negative Breast Cancer: A 2D and 3D *in vitro* validation**

Tiago P. Ribeiro^a,b,c,d,*^, Francisca L. Gomes^e,f^, Rui Vilarinho^g^, Christiane Salgado^a,b^, Maria Cristina L. Martins^a,b,h^, Joaquim Agostinho Moreira^g^, Fernando J. Monteiro^a,b,c,d^, Marta S. Laranjeira^a,b,d,*^

^a^ i3S-Instituto de Investigação e Inovação em Saúde, Universidade do Porto, Rua Alfredo Allen 208, 4200-135 Porto, Portugal

^b^ INEB-Instituto de Engenharia Biomédica, Universidade do Porto, Rua Alfredo Allen 208, 4200-135 Porto, Portugal

^c^ FEUP-Faculdade de Engenharia, Universidade do Porto, Rua Dr. Roberto Frias, s/n 4200-465, Porto, Portugal

^d^ Porto Comprehensive Cancer Center Raquel Seruca (P.CCC), R. Dr. António Bernardino de Almeida, 4200-072 Porto, Portugal

^e^ Department of Molecules and Materials Laboratory of Biointerface Chemistry, Faculty of Science and Technology Technical Medical Centre and MESA+ Institute University of Twente Drienerlolaan 5, Enschede 7522NB, The Netherlands

^f^ Department of Developmental BioEngineering Leijten Laboratory Faculty of Science and Technology Technical Medical Centre University of Twente Drienerlolaan 5, Enschede 7522NB, The Netherlands

^g^ IFIMUP- Instituto de Física de Materiais Avançados, Nanotecnologia e Fotónica, Departamento de Física e Astronomia, Faculdade de Ciências, Universidade do Porto, Rua do Campo Alegre, s/n- 4169-007 Porto, Portugal.

^h^ ICBAS - Instituto de Ciências Biomédicas Abel Salazar, Universidade do Porto, Rua de Jorge Viterbo Ferreira, 4050-313, Porto, Portugal


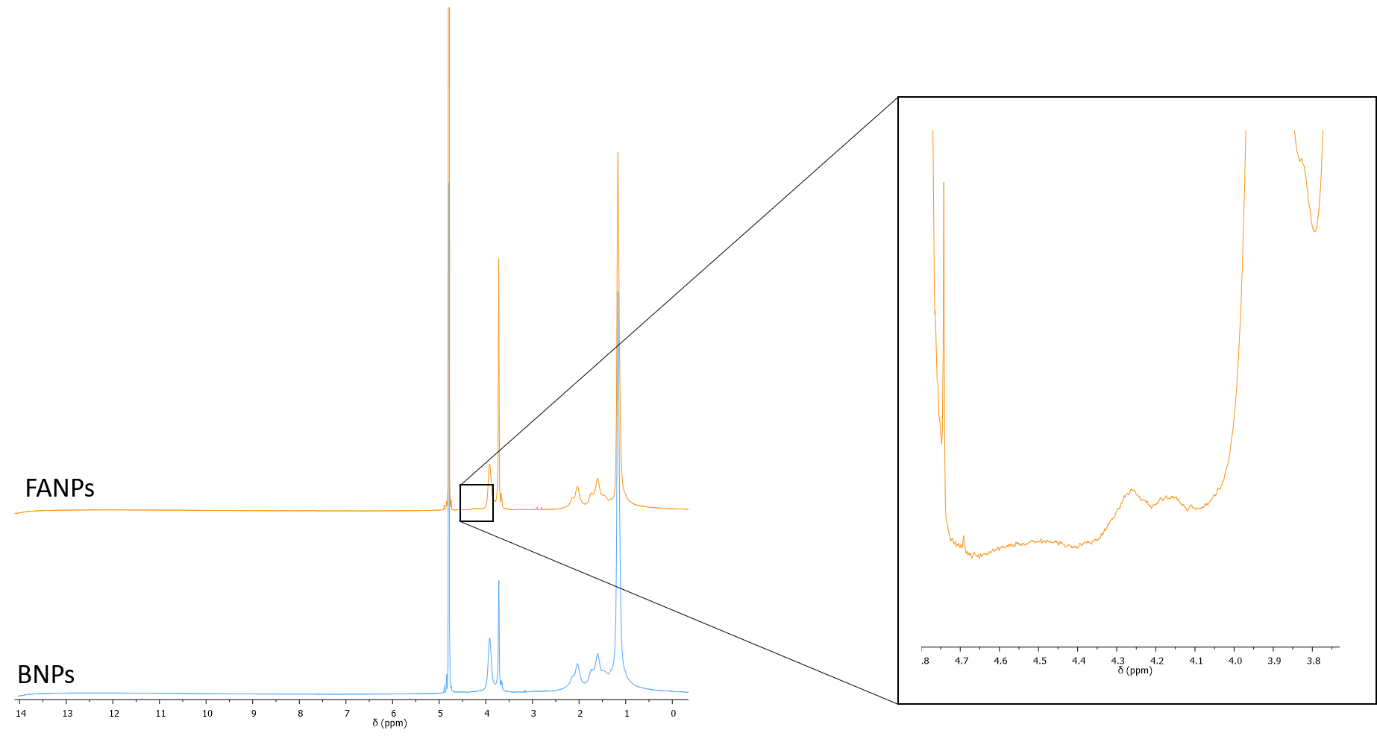


**Fig. S1 –** ^1^H NMR spectra of BNPs and FANPS in D_2_O at 25 °C. Insert: Characteristic peaks of folic acid.


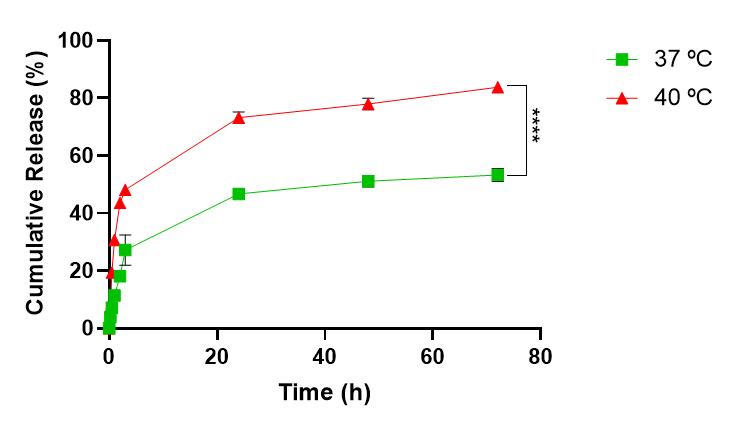


**Fig. S2 -** Drug release profile of BNPs, in PBS, at 37 and 40 °C.


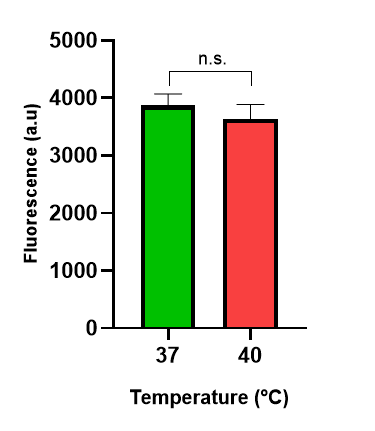


**Figure S3 –** Metabolic activity of MDA-MB-468 spheroids at 37 and 40 °C after 72 h of culture.

**Table S1 –** Summary of FTIR peaks and corresponding functional groups

| **Peak wavenumber (cm^-1^)** | **Chemical group** |
| --- | --- |
| 1106 | C-O |
| 1250 | C-O-C |
| 1371 | C-H (deformation) |
| 1384 | C-H (deformation) |
| 1460 | C-H (deformation) |
| 1548 | C-N |
| 1640 | C=O |
| 1718 | C=O |
| 2875 | C-H (stretching) |
| 2975 | C-H (stretching) |
| 3299 | O-H |
| 3500 | N-H |

**Table S2** – Summary RAMAN peaks and corresponding functional groups

| **RAMAN shift (cm^-1^)** | **Chemical group** |
| --- | --- |
| 680 | C=C |
| 700 | C=C |
| 840 | C-C (stretching from monomers) |
| 920 | C-C (stretching from backbone) |
| 950 | C-C (stretching from backbone) |
| 1125 | CH_3_ (rocking) |
| 1165 | C-O-C (stretching) |
| 1390 | CH_3_ (symmetric stretching) |
| 1450 | C-H (bending) |
| 1640 | C-N (stretching) |
| 2870 | CH_3_ (symmetric stretching) |
| 2920 | CH_2_ (symmetric stretching) |
| 2970 | CH_3_ (antisymmetric stretching) |
